# Phytochemical Analysis, Antibacterial Activity and Modulating Effect of Essential Oil from *Syzygium cumini* (L.) Skeels

**DOI:** 10.3390/molecules27103281

**Published:** 2022-05-20

**Authors:** Priscilla Augusta de Sousa Fernandes, Raimundo Luiz Silva Pereira, Antonia Thassya Lucas dos Santos, Henrique Douglas Melo Coutinho, Maria Flaviana Bezerra Morais-Braga, Viviane Bezerra da Silva, Adrielle Rodrigues Costa, Maria Elizete Machado Generino, Maraiza Gregorio de Oliveira, Saulo Almeida de Menezes, Luciano Temoteo dos Santos, Abolghasem Siyadatpanah, Polrat Wilairatana, Tainá Machado Aguiar Portela, Ma Aparecida Barbosa Ferreira Gonçalo, José Weverton Almeida-Bezerra

**Affiliations:** 1 Laboratório de Micologia Aplicada do Cariri (LMAC), Universidade Regional do Cariri–URCA, Av. Cel Antônio Luis, 1161, Pimenta, Crato 63105-010, Brazil; priscilla.fernandes@urca.br (P.A.d.S.F.); thassyalucas@hotmail.com (A.T.L.d.S.); flavianamoraisb@yahoo.com.br (M.F.B.M.-B.); adrielle.arc@hotmail.com (A.R.C.); maria.machado@urca.br (M.E.M.G.); luciano.temoteosantos@gmail.com (L.T.d.S.); aparecida.barbosa@urca.br (M.A.B.F.G.); weverton.almeida@urca.br (J.W.A.-B.); 2 Laboratório de Microbiologia e Biologia Molecular (LMBM), Universidade Regional do Cariri–URCA, Av. Cel Antônio Luis, 1161, Pimenta, Crato 63105-010, Brazil; raimundoluizbio@gmail.com; 3 Departamento de Botânica, Universidade Federal de Pernambuco–UFPE, Av. Prof. Moraes Rego, 1235, Recife 50670-901, Brazil; viviane.silva@urca.br; 4 Departamento de Ciências Biológicas, Universidade Federal do Cariri–UFCA, Av. Ten. Raimundo Rocha, 1639-Cidade Universitária, Juazeiro do Norte 63048-080, Brazil; maraaiza0104@hotmail.com; 5 Centro de Biotecnologia, Universidade Federal do Rio Grande do Sul–UFRGS, Porto Alegre 91501-970, Brazil; saulomenezes99@gmail.com; 6 Ferdows School of Paramedical and Health, Birjand University of Medical Sciences, Birjand 9717853577, Iran; 7 Department of Clinical Tropical Medicine, Faculty of Tropical Medicine, Mahidol University, Bangkok 10400, Thailand; 8 Faculdade de Medicina, Universidade Nilton Lins, Parque das Laranjeiras, Av. Prof. Nilton Lins, 3259-Flores, Manaus 69058-030, Brazil; taina_portela@hotmail.com

**Keywords:** *Eugenia jambolana*, α-pinene, bioactivity, nerol, Myrtaceae

## Abstract

One of the main global problems that affect human health is the development of bacterial resistance to different drugs. As a result, the growing number of multidrug-resistant pathogens has contributed to an increase in resistant infections and represents a public health problem. The present work seeks to investigate the chemical composition and antibacterial activity of the essential oil of *Syzygium cumini* leaves. To identify its chemical composition, gas chromatography coupled to mass spectrometry was used. The antibacterial activity test was performed with the standard strains *Escherichia coli* ATCC 25922, *Pseudomonas aeruginosa* ATCC 25853 and *Staphylococcus aureus* ATCC 25923 and multidrug-resistant clinical isolates *E. coli* 06, *P. aeruginosa* 24 and *S. aureus* 10. The minimum inhibitory concentration (MIC) was determined by serial microdilution as well as the verification of the modulating effect of the antibiotic effect. In this test, the oil was used in a subinhibitory concentration. The test reading was performed after 24 h of incubation at 37 °C. The results show that the major chemical constituent is α-pinene (53.21%). The oil showed moderate activity against *E. coli* ATCC 25922, with the MIC of 512 µg/mL; there was no activity against the other strains. The oil potentiated the effect of antibiotics demonstrating possible synergism when associated with gentamicin, erythromycin and norfloxacin against *E. coli* 06 and *S. aureus* 10.

## 1. Introduction

Bacteria are ubiquitous, and many of them are pathogenic. The entry of these microorganisms and their recognition by the host defense system induce stress in host cells [1]. Over time, bacteria have also developed strategies, involving resistance to host defense mechanisms as well as some used antibacterial therapy [1].

One of the main global problems that affect human health is the development of bacterial resistance to different drugs. Thus, the growing number of multidrug-resistant pathogens has contributed to an increase in resistant infections and represents a serious problem [2].

In this context, the inefficiency of antibiotics and antimicrobial resistance have been a huge challenge and problem faced by medicine. In view of this, many studies have been attributed to the search for new agents with antibacterial action, including natural products from medicinal plants or of microbial origin [3,4,5,6].

In this scenario, studies of natural products with emphasis on isolation, purification and characterization of secondary metabolites of medicinal plants have significantly impacted the discovery of substances with antimicrobial action since these secondary metabolites have shown relevant benefits in the treatment of numerous diseases [7,8,9].

Essential oils (EOs) present several volatile compounds with expressive antimicrobial activity [9,10,11]. With the considerable increase in antimicrobial resistance, these natural products have proven to be efficient weapons against multiresistant microorganisms [10].

The Myrtaceae family includes around 150 genera and 3600 species with a cosmopolitan distribution [12]. Studies with EOs of different species of the family have been carried out, for example, we can mention biological and pharmacological activities such as antiparasitic [13], antibacterial [14], antifungal [15], antioxidant [16] and many more. *Syzygium cumini* is used in folk medicine to treat leucorrhea, urination pain, dermatitis and the presence of bloody diarrhea [17] and has been investigated for various biological activities such as hepatoprotective effect, antimicrobial, anti-inflammatory, hypoglycemic, hepatoprotective and hypolipidemic activity [18]. Given the above, the present work aimed to investigate the chemical composition of the EO from leaves of *S. cumini* and also its antibacterial activity and the ability to potentiate the action of antibiotics against some resistant bacterial strains.

## 2. Results and Discussion

The essential oil from the leaves of *S. cumini* presented a total of 18 chemical constituents, constituting 96.62% of its composition (Table 1). Among the chemical constituents identified by GC–MS, the monoterpene α-pinene (53.21%) was the major compound corresponding to half of the chemical composition of the EO. In addition to it, another monoterpene that stood out was nerol (9.38%) as a secondary constituent.

It is noted in Table 1 that there was a variation in the chemical composition of the EO of *S. cumini* reported in some works, where the major compound, α-pinene, had lower percentages, for example, 0.49 [18], 21.5% [20], 31.85% [21] and 32.32% [22]. Common and different constituents were also recorded [18,20,21,22,23]. There was also variation in the total percentage of compounds identified; in this research, it corresponded to 96.62%; values ranging from 95.3% [20], 98.28% [22] and 99.29% [21] were identified. This variation may have been due to differences between the collection sites as biotic and abiotic factors, as well as the collection period, can influence the composition of the oil [24].

In other species of the same genus, α-pinene is also the main constituent, e.g., in *Syzygium polyanthum* (Wight) Walp. where the compound is present in 38.46% of the EO [25]. In *Syzygium aromaticum* (L.) Merr. & L.M.Perry, the major compound is eugenol with the concentration ranging from 50% to 82.4% [26,27,28]. In other Myrtaceae species, α-pinene is also one of the main constituents, such as in *Callistemon citrinus* (Curtis) Skeels (35.1%) and *Baekea frutescens* L. (11.1%) [29], *Myrtus communis* L. (35.8%) [30] and *Campomanesia xanthocarpa* (Mart.) O.Berg (15%) [31]. This may indicate that this compound (α-pinene) is a phytochemical marker of the family.

According to Table 2, against the standard strains the EOSC showed moderate activity against *E. coli* ATCC 25922, with a MIC of 512 µg/mL. Against *P. aeruginosa* ATCC 25853 and *S. aureus* ATCC 25923, there was no activity; for both, the MIC was ≥ 1024 µg/mL. The EOSC did not show antibacterial activity against multidrug-resistant strains.

*S. cumini* is a species with widespread use in folk medicine to treat skin diseases and dysentery and improve healing processes [32]. Previous studies documented antimicrobial activity of the seed extract and fruit pulp [33]. The antibacterial effect observed against *E. coli* can be attributed to a synergistic action of the constituents, and not only of the major constituent. In a previous study, when such a constituent was tested as a single compound against the same strain of *E. coli*, its low activity was observed [34].

Other researches have registered inhibitory and bactericidal activity of the EOSC against *S. aureus* [35], the species was also susceptible to the extract of the seeds of *S. cumini* [36]. Against *E. coli,* the EOSC showed low inhibition when tested previously [37]. There was growth inhibition when the EOSC was tested against *P. aeruginosa* [38].

Other Myrtaceae species have been tested for antibacterial activity. The EO of *Myrtus communis* L. showed good inhibitory and bactericidal activity against *E. coli* and other bacterial strains [30]. *Syzygium aromaticum* oil showed strong inhibitory activity against *S. aureus* [39] and other Gram-positive and Gram-negative strains [26], as well as *Melaleuca racteate* F.Muell. and *Melaleuca alternifolia* Cheel [40].

**Figure 1:**
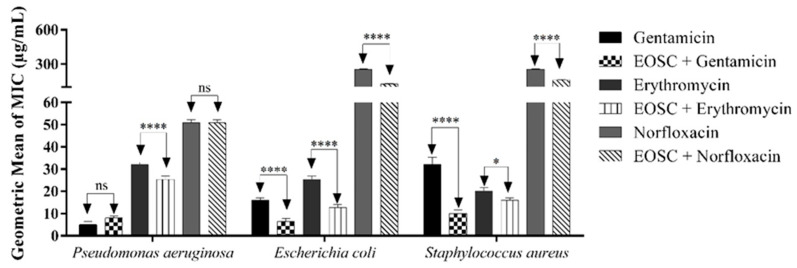
Ability of the EOSC at a subinhibitory concentration of 128 μg/mL (MIC/8) to potentiate the action of antibiotics against multidrug-resistant bacterial strains. * = 0.05; **** = 0.0001; ns = non-significant.

The EO was able to significantly modify the action of antibiotics as shown in Figure 1, except against *P. aeruginosa* 24, with no significance when the EOSC was combined with gentamicin and norfloxacin, demonstrating a possible antagonism with the former. This may have occurred because this species is Gram-negative and has an efflux pump [41]; these pumps contribute to resistance to antibiotics and antimicrobial compounds [42]. However, this needs to be verified in the future. Against *E. coli* 06 and *S. aureus* 10, the oil associated with the antibiotics gentamicin, erythromycin and norfloxacin demonstrated possible synergism, potentiating their action against multidrug-resistant strains.

Essential oils act by several mechanisms of action against bacterial cells. In this research, it was possible to verify the modification of the action of antibiotics and the oil possibly acted through synergism, which can be verified through the checkerboard test in future research, potentiating their action against both Gram-negative and Gram-positive strains, the mechanism of action involved may be interference with the wall proteins that are involved in the transport of substances into the cell [43], thus facilitating the action of the antibiotic.

## 3. Materials and Methods

### 3.1. Plant Material and Collection License

**Figure 2:**
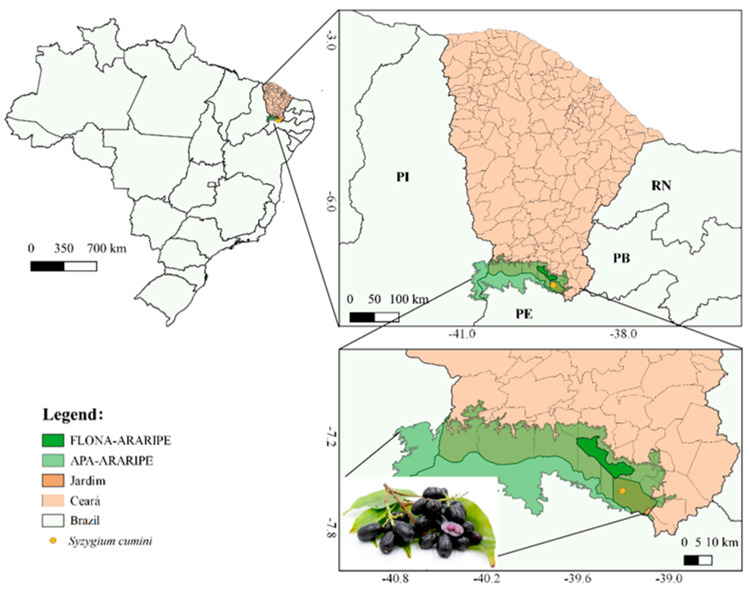
Location map of *Syzygium cumini* leaves collection in Serra Gravatá, municipality of Jardim, Ceará, Brazil.

The leaves of *S. cumini* were collected in Serra Gravatá, municipality of Jardim, Ceará, Brazil, at 7°33′18″ W, 39°18′23″ S (Figure 2). The exsiccate was deposited in Herbário Caririense Dárdano de Andrade-Lima with voucher No. 13593. For collection, authorization was obtained from the Biodiversity Authorization and Information System, SisBio, under No. 64011-1. The research was also registered in the National System for the Management of Genetic Heritage and Associated Traditional Knowledge (SisGen) under registration No. A7AEBD7.

### 3.2. Essential Oil Extraction

After collection, the leaves were dried at room temperature and manually crushed to increase the contact surface with the extraction solvent. Then, they were placed in a 5 L glass flask containing 2 L of distilled water and subjected to constant boiling for two continuous hours to collect the EO in a Clevenger system; 200 g of leaves were used in each extraction, totaling 1200 g. A yield of 0.159% was obtained, and the oil was stored in an amber bottle and refrigerated at −4 °C until chemical characterization and tests were carried out.

### 3.3. Chemical Composition of Essential Oil

#### 3.3.1. Gas Chromatography

The essential oil of *S. cumini* after preparation was submitted to GC analysis in a Varian 3800 Gas Chromatograph (International Equipment Trading Ltd., Mundelein, IL, USA) equipped with a capillary fused silica column (25 m × 0.25 mm) coated with SE-54. The GC conditions used were as follows: carrier gas He (1 mL/min); column injector temperature, 200 °C; flame ionization detector (FID) temperature, 250 °C; column temperature, from 60 °C to 325 °C at 4 °C/min. GC–MS analyses were performed on an HP 5973-6890 GC-MSD system operating in the EI mode at 70 eV, equipped with an HP-5 crosslinked capillary column (30 m × 0.25 mm). The temperature of the column and the injector were the same as those from GC [44].

#### 3.3.2. Identification of the Components

Identification of the constituents of the *S. cumini* essential oil was based on the retention index (RI), determined with reference to the homologous series of *n*-alkanes, C_7_–C_30_, under identical experimental conditions, comparing with the mass comparison of the mass spectra with those of NBS Library and those described by Adams [45]. The relative amounts of individual components were calculated based on the CG peak area (FID response).

### 3.4. Antibacterial Activity

#### 3.4.1. Bacterial Strains, Culture Media and Drugs

For the antibacterial activity tests, standard bacterial strains and bacterial clinical isolates were used. To determine the minimum inhibitory concentration (MIC), the standard strains were used: *Escherichia coli* ATCC 25922, *Pseudomonas aeruginosa* ATCC 25853 and *Staphylococcus aureus* ATCC 25923. The clinical isolates were as follows: *E. coli* 06, *P. aeruginosa* 24 and *S. aureus* 10, with multidrug resistance [46] (Table 3).

After the growth period, samples from the respective colonies were diluted in test tubes containing 3 mL of sterile saline solution (NaCl 0.9%). The suspensions were shaken in a vortex device and their turbidity was compared and adjusted to the 0.5 McFarland scale (1.5 × 10^8^ colony-forming units/mL) [47].

For antibacterial assays, the Brain Heart Infusion (BHI, Merck KGaA, Darmstadt, Germany) culture medium was used, which was prepared according to the measures recommended by the manufacturer. The drugs used to evaluate the modulating capacity of its effect using the essential oil of *S. cumini* (EOSC) were gentamicin (aminoglycosides), erythromycin (macrolides) and norfloxacin (fluoroquinolone) (Cimed, Porto Alegre, Brazil).

The natural product (10 mg) was diluted in 1 mL of dimethyl sulfoxide (DMSO, Merck KGaA, Darmstadt, Germany) and 8765 μL of sterile distilled water so that the respective solutions reached a concentration of 1024 μg/mL. Antibiotics were diluted following the same method, although in sterile distilled water [9]. The test was performed in triplicate.

#### 3.4.2. Minimum Inhibitory Concentration (MIC)

The MIC, the minimum concentration responsible for totally inhibiting bacterial growth, was determined according to the methodology used by Gomes et al. [48]. A 1000 μL solution containing 100 μL of an inoculum and 900 μL of a liquid culture medium (10% BHI) was prepared in Eppendorf tubes. This solution was distributed among 96-well plates filled numerically by adding 100 μL to each well. Subsequently, 100 μL of the EOSC were added to the first well and serially microdiluted; the concentrations varied from 0.5 to 512 μg/mL; then, the plates were incubated for 24 h at 37 °C.

After this incubation period, 20 μL of a resazurin solution were added to each well so that oxidoreductive reactions occurred where there was still bacterial growth. After one hour, the color change of the wells was analyzed, where a change from blue to red corresponded to microbial growth, and what remains blue means the absence of growth.

#### 3.4.3. Drug-Modifying Effect

To evaluate the potentiating capacity of the EOSC, the methodology proposed by Coutinho et al. [49] was used, in which after MIC tests with resistant bacteria, the results were used to determine the subinhibitory concentrations (MIC/8) to be used with antibiotics at concentrations ranging from 0.1 to 512 μg/mL. Thus, for the tests, 1162 μL of 10% BHI were used, with 150 μL of the inoculum of each strain and the natural product with a volume corresponding to a subinhibitory concentration, while the control group was prepared with only 1350 μL of BHI (10%) and 150 µL of a bacterial suspension. Subsequently, serial microdilution was performed with the antibiotic (0.5 to 512 µg/mL), being performed with 100 µL of each drug until the penultimate well. The plates were incubated (24 h at 37 °C) and read with the addition of 20 μL of resazurin according to the MIC reading. The test was performed in triplicate.

### 3.5. Statistical Analysis

The results were analyzed using GraphPad Prism version 6 (Graph Pad Software Inc., San Diego, CA, USA), using one-way ANOVA followed by Tukey’s post-hoc test and were considered significant when *p* < 0.05.

## 4. Conclusions

*Syzygium cumini* is a species of wide popular use in Brazil. As for its chemical composition, it was possible to identify it almost completely, with α-pinene being the major constituent as well as in other species of the same genus and family. Despite the wide ethnopharmacological use for the treatment of infectious and parasitic diseases, its oil did not show antibacterial activity. However, when associated with antibiotics, it was able to intensify the action of the drugs, which may indicate that the natural product may be able to synergistically modify the action of antibiotics. Thus, further investigations with oil collected at other times of the year and in other parts of Chapada do Araripe are necessary to better understand the chemical composition of the species in the region, as well as elucidate the mechanisms and key compounds involved in modifying the action of antibiotics.

## Figures and Tables

**Table 1:** Chemical composition of the essential oil from leaves of *Syzygium cumini* (L.) Skeels.

Compounds	RI ^a^	RI ^b^	%
α-pinene	937	939	53.21
β-pinene	979	981	3.01
β-myrcene	995	991	0.75
Limonene	1029	1031	1.25
Nonalol	1105	1103	5.62
Linalool	1099	1098	3.98
α-terpineol	1187	1189	2.09
Tetradecane	1226	1221	0.27
Nerol	1228	1228	9.38
(E,Z)-2,4-decadienal	1296	1295	0.98
Geranil acetate	1385	1384	3.43
Ionone	1387	1387	1.29
Damascone	1409	1411	1.08
Caryophyllene	1417	1418	2.81
α-humulene	1451	1452	1.57
Nerolidol	1569	1564	5.73
Globulol	1581	1583	0.06
α-cadinol	1646	1649	0.11
Total identified (%)			96.62

Source: Research data; ^a^ experimental retention index (based on the n-alkane C_7_–C_30_ homologous series); ^b^ literature retention index [19].

**Table 2:** Minimum inhibitory concentration (µg/mL) of the *S. cumini* EO against the standard multidrug-resistant strains.

Strains	*E. coli*	*P. aeruginosa*	*S. aureus*
Standard strains	ATCC 25922	ATCC 25853	ATCC 25923
	512	≥1024	≥1024
Multidrug-resistant strains	EC06	PA24	SA10
EOSC	≥1024	≥1024	≥1024
Gentamicin	18	4	35
Erythromycin	25	32	23
Norfloxacin	290	56	300

**Table 3:** Antibiogram of the strains used in the MIC and modulation tests with their antibiotic resistance profile and origin.

Bacterial Strain	Source	Resistance Profile
*Escherichia coli* 06	Urine culture	Cephalexin, cefoxitin, cefadroxil, ceftriaxone, cefepime, ampicillin/sulbactam
*Pseudomonas aeruginosa* 24	Urine culture	Amikacin, imipenem, ciprofloxacin, levofloxacin, piperacillin/tazobactam, ceftazidime, merpenem, cefepime
*Staphylococcus aureus* 10	Rectal swab culture	Cefadroxil, cephalexin, cephalothin, oxacillin, penicillin, ampicillin, amoxicillin, moxifloxacin, ciprofloxacin, levofloxacin, ampicillin/sulbactam, amoxilin/clavulanic acid, erythromycin, clarithromycin, azithromycin, clindamycin

## Data Availability

Not applicable.

## Sample Availability

Samples of the compounds are available from the authors.
